# Remodeling the Inflammatory Microenvironment: Nanomaterial‐Based Targeted Strategies for Systemic Lupus Erythematosus and Lupus Nephritis

**DOI:** 10.1002/smsc.202500549

**Published:** 2026-03-08

**Authors:** Cheng Zhou, Jiayi Li, Jian Zhang, Haifeng Wang, Haitao Lu, Shunlai Shang, Wenge Li

**Affiliations:** ^1^ Department of Nephrology China‐Japan Friendship Hospital Beijing China

**Keywords:** immune cell targeted nanomedicines, lupus nephritis, nanoparticle drug delivery systems, renal intrinsic cells targeted nanomedicines, systemic lupus erythematosus

## Abstract

Systemic Lupus Erythematosus (SLE) is a complex autoimmune disorder, with Lupus Nephritis (LN) representing a severe complication that affects a significant proportion of patients. Current treatments, including corticosteroids and immunosuppressants, are limited by systemic toxicity and nonspecific biodistribution. Nanomaterial‐based drug delivery systems have emerged as a promising strategy to overcome these challenges by improving drug solubility, facilitating targeted delivery, and reducing off‐target effects. This review comprehensively discusses the rational design and application of advanced nanomaterials—such as liposomes, polymeric nanoparticles, dendrimers, and biomimetic nanocarriers—in the context of SLE/LN therapy. It highlights how tailored nanoplatforms can selectively target key immune cells (e.g., T cells, B cells, macrophages, and dendritic cells) and renal parenchymal cells, support combination therapy, and improve therapeutic outcomes while minimizing off‐target effects. Furthermore, the review critically examines current challenges and future prospects for clinical translation, advocating for smarter nano‐therapeutics capable of integrating immune modulation and organ‐specific targeting. This work aims to bridge materials design and immunology, providing insights into next‐generation treatments for SLE/LN diseases.

## Graphical abstract

Current treatments for severe lupus nephritis are limited by side effects due to poor drug targeting. This review explores how smart nanomaterials—like liposomes and biomimetic carriers—can precisely deliver drugs to overactive immune cells and the kidneys. These nanotherapeutics enhance efficacy, reduce toxicity, and enable combination strategies. The discussion bridges materials science and immunology to outline a promising path toward next‐generation, targeted therapies.

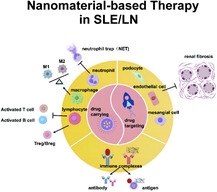

## 1 Introduction

Systemic Lupus Erythematosus (SLE) is a chronic autoimmune disease characterized by dysregulation of the immune system and diverse clinical manifestations that can affect multiple organs, including the skin, joints, serous membranes, nervous system, and kidneys. Among these, lupus nephritis (LN) is one of the most common and serious complications [1]. Approximately 40%–75% of SLE patients progress to LN, which is marked by persistent immune‐mediated inflammation within the kidneys, ultimately leading to renal fibrosis and functional failure, significantly impairing patient prognosis [2]. The pathogenesis of SLE/LN involves multiple dysregulations of both innate and adaptive immunity, including abnormal activation of autoreactive T and B cells, production of autoantibodies, and release of large amounts of inflammatory cytokines [3, 4].

Current clinical management of SLE/LN primarily relies on nonspecific anti‐inflammatory and immunomodulatory agents such as glucocorticoids, immunosuppressants, and biologics [5]. However, due to their systemic distribution and lack of targeting specificity, these drugs often cause severe side effects, including secondary infections, metabolic disorders, and multiorgan toxicity, which limit their long‐term use [6]. Therefore, developing novel formulations and drug delivery systems that can precisely target pathogenic cells or inflammatory sites, enhance therapeutic efficacy, and reduce systemic exposure has become a major focus of current research [7]. In recent years, the rapid advancement of nanomedicine has provided new strategies for the treatment of SLE/LN. Nanomaterials, by virtue of their size effects, surface modifiability, and good biocompatibility, serve as ideal drug carriers enabling targeted delivery and controlled release [8, 9, 10]. It is particularly noteworthy that certain nanomaterials can be actively taken up by innate immune cells (such as macrophages and dendritic cells) or overactivated resident cells in the kidneys, thereby specifically accumulating at disease sites, enhancing drug distribution in affected tissues, and reducing off‐target side effects. Various nanoplatforms—including liposomes, polymeric nanoparticles, dendrimers, and inorganic nanomaterials—have been widely used to deliver drugs with different mechanisms of action, demonstrating excellent therapeutic potential in experimental models of SLE/LN [11, 12, 13, 14]. Recent advances have further refined these strategies for better target ability. For instance, biomimetic nanocarriers (e.g., cell membrane‐coated nanoparticles, engineered exosomes) leverage inherent biological interfaces and “homing” properties for more precise active targeting [15, 16]. Meanwhile, stimuli‐responsive platforms (e.g., those sensitive to pathological pH, reactive oxygen species, or specific enzymes) enable on‐demand drug release, enhancing the intelligence of therapeutic intervention [17, 18]

Previous reviews on nanomaterials in autoimmune diseases have often adopted an overly broad scope, attempting to cover a wide range of conditions such as rheumatoid arthritis, multiple sclerosis, and diabetes. This broad approach has typically resulted in superficial or limited discussions of the complex disease SLE/lupus nephritis (SLE/LN), failing to provide an in‐depth analysis of its unique immunopathology and the specific challenges of renal targeting [19, 20]. To address these limitations, this review for the first time establishes a highly focused and deeply integrated analytical framework. We concentrate exclusively on SLE/LN, systematically linking disease‐specific immunopathogenesis with the rational design and functionalization strategies of nanomaterials. This framework extends to precise targeting interventions against specific immune cell subsets (e.g., pathogenic B cells, M1 macrophages) and renal intrinsic cells (e.g., podocytes, mesangial cells). Finally, the current challenges and clinical translation prospects of such nanoformulations will be reviewed and discussed, with the aim is to provide insights, grounded in a fundamental understanding of the disease and extending through material design to clinical translation, for the development of next‐generation nanotherapeutic agents that are highly selective, efficacious, and safe for SLE/LN.

## 2 Immunomodulatory Mechanism of SLE/LN

SLE is a quintessential autoimmune disorder characterized by the generation of a diverse array of autoantibodies and ICs. This process entails the activation of the complement system and the subsequent recruitment of inflammatory cell infiltration within the targeted organs, ultimately resulting in both acute and chronic inflammatory responses across multiple systems. The cardinal pathological hallmarks of SLE encompass inflammation, IC deposition, vasculitis, and vasculopathy, which frequently affect an extensive range of organs and tissues, encompassing the kidneys, lungs, vasculature, skin, and even the central nervous system [21, 22]. In SLE, autoreactive T cells and B cells, typically quiescent, become activated and escape the regulatory constraints of peripheral tolerance mechanisms [23, 24]. B cells exhibit hyperreactivity, resulting in the generation of a spectrum of autoantibodies, the formation of ICs, and the induction of inflammation across various organs and tissues. Apart from the intrinsic hyperactivity of B cells, SLE patients frequently exhibit aberrant T cell immune responses. Notably, the activation and proliferation of pathogenic T helper 1 (Th1) cells, T helper 17 (Th17) cells, and TCR‐*αβ*+ CD4‐CD8‐ double‐negative (DN) T cells are prominently observed. Furthermore, the immune responses mediated by regulatory CD4+ and CD8+ T lymphocytes become deregulated, accompanied by excessive production of T cell cytokines and aberrant cross‐talk between B cells and T cells [25]. Innate immune cells, including macrophages, neutrophils, and dendritic cells, particularly their roles in the clearance of apoptotic debris, antigen presentation, and the secretion of inflammatory cytokines, also play pivotal roles in the pathogenesis of SLE [26, 27]. A diverse array of autoantibodies, including anti‐double‐stranded DNA (anti‐dsDNA), anti‐single‐stranded DNA (anti‐ssDNA), anti‐RNA, and anti‐phospholipid antibodies, can be detected in SLE. These autoantibodies subsequently form ICs, which deposit in multiple organs, thereby eliciting a spectrum of clinical manifestations [28].

**FIGURE 1:**
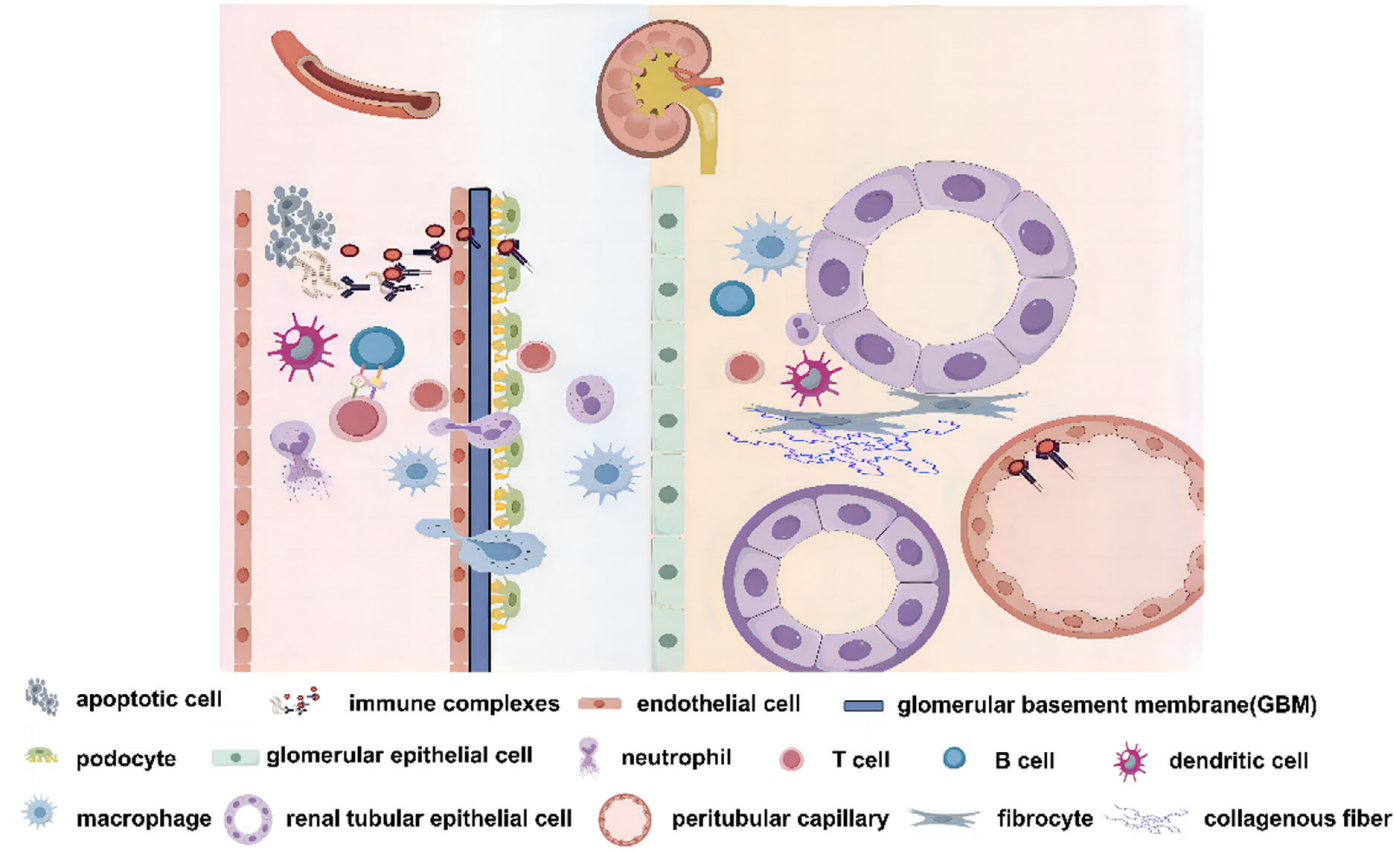
Immunomodulatory mechanism of SLE/LN. Apoptotic cells liberate their cellular contents, encompassing nuclear antigens like DNA and RNA, into cellular debris, which possesses the capacity to elicit immune system stimulation and facilitate the initiation of SLE. Innate immune cells, including macrophages, neutrophils, and dendritic cells, perceive and respond to apoptotic debris, thereby driving the activation of immune cells. Activated T cells exhibit hyperproduction of cytokines and elicit aberrant B‐T cell interactions. Subsequently, activated B cells generate autoantibodies that bind to autoantigens, forming ICs. These ICs accumulate within the glomerular endothelium, podocytes, tubular interstitium, and blood vessels, thereby triggering immune cell infiltration. This process exacerbates local renal inflammation and ultimately progresses to renal fibrosis.

Lupus nephritis (LN) represents a quintessential manifestation of SLE. Approximately 25%–50% of individuals diagnosed with SLE exhibit LN, a condition distinguished by the deposition of ICs within glomeruli and renal tubules, accompanied by the recruitment and activation of inflammatory cells [2]. This process ultimately results in renal tissue damage and compromised kidney function (Figure 1). SLE can elicit the production of autoantibodies that specifically bind to renal autoantigens, including antibodies targeted against double‐stranded DNA (dsDNA) and antinuclear antibodies (ANA). These autoantibodies subsequently form ICs within the kidney tissue [29]. The deposition of these ICs disrupts immune homeostasis and induces renal pathology in the affected renal regions. Inflammation constitutes a pivotal factor in the pathogenesis of lupus nephritis, and the inhibition of inflammatory processes emerges as a potentially promising therapeutic strategy. The localized inflammatory response within the kidney, predominantly initiated and exacerbated by innate immune cells, particularly monocytes and notably macrophages, plays a crucial role in the inflammatory cascade [30, 31]. The proliferation and activation of B cells exhibit a tight correlation with the progression of lupus nephritis (LN), wherein autoreactive B cells and plasma cells (PCs) occupy a pivotal role in the disruption of systemic immune tolerance and the induction of localized autoimmune inflammation, ultimately culminating in renal damage [32, 33]. Furthermore, the kidneys of patients with SLE demonstrate extensive infiltration by both CD4+ and CD8+ T cell lineages. Abnormalities in the adaptive immune system in lupus nephritis (LN) exert profound effects on T‐cell responsiveness, cytokine production by T cells, and the intricate B‐T cell interplay [34, 35]. Furthermore, beyond immune cells, a diverse array of intrinsic renal tissue cells contributes to the progression of LN, encompassing mesangial cells, podocytes, glomerular endothelial cells (GECs), and tubular epithelial cells [36]. Podocyte injury and the disruption of their fusion exacerbate proteinuria [37]. Notably, excessive proliferation of mesangial cells (MCs) precipitates renal fibrosis, ultimately culminating in glomerular sclerosis and renal failure [38].

## 3 Current Status and Limitations of SLE/LN

Currently, the most prevalent treatment strategies for SLE encompass a spectrum of pharmacological agents, including nonsteroidal anti‐inflammatory drugs (NSAIDs), antimalarial drugs, oral corticosteroids, cytotoxic agents, and immunosuppressants [5]. However, a significant limitation of these contemporary therapeutic approaches lies in their partial efficacy. This is attributed to the fact that these medications often exert their effects by inhibiting not only the pathological cells implicated in SLE pathogenesis but also the cells that are essential for regulating and controlling the activities of those pathological cells. Furthermore, the widespread immunosuppressive actions of pharmacological and/or biological agents are frequently accompanied by detrimental toxic side effects [6, 7]. During acute exacerbations of Lupus Nephritis (LN), high‐dose immunosuppressive medications are primarily administered; however, the therapeutic efficacy achieved remains suboptimal.

NSAIDs are often efficacious in alleviating mild symptoms associated with SLE, including joint pain, musculoskeletal discomfort, fever, headache, and mild serositis. However, they can induce renal side effects, such as sodium retention and decreased glomerular filtration rate [39]. Notably, Lupus Nephritis (LN) constitutes a risk factor for acute renal failure induced by NSAIDs. Consequently, patients undergoing long‐term NSAID therapy require rigorous monitoring for signs of renal or hepatic damage and bleeding [40]. For many years, glucocorticoids (GCs) have been regarded as the most potent therapeutic agent for the treatment of SLE, owing to their potent anti‐inflammatory and immunosuppressive properties. However, their utilization is constrained by the necessity for high dosages, which is attributed to their unfavorable pharmacokinetic profiles and biological distribution characteristics. However, most cells in tissues throughout the body express GC receptors, resulting in glucocorticoids potentially exerting unintended effects on nontarget organs. Specifically, long‐term or high‐dose glucocorticoid (GC) therapy is intricately linked to a multitude of adverse effects, including Cushing's syndrome, hypertension, peptic ulcers, and osteoporosis, thereby necessitating caution in administering prolonged or high‐dose GC regimens. Antimalarial drugs, particularly chloroquine‐based antimalarials such as chloroquine and hydroxychloroquine, are instrumental in maintaining disease remission, inhibiting the onset of disease, and mitigating damage to the kidney and central nervous system. However, these agents also exhibit off‐target side effects, encompassing gastrointestinal discomfort, skin reactions, and headaches. Consequently, their use often necessitates combination therapy to balance therapeutic efficacy and adverse event profile [41].

Cytotoxic agents, including azathioprine, mycophenolate mofetil (MMF), cyclophosphamide, cyclosporine, and methotrexate, have demonstrated significant clinical efficacy in inducing and maintaining remission in a substantial proportion of SLE patients [42]. However, their therapeutic utility is often constrained by off‐target tissue distribution, dose‐limiting toxicities (e.g., nephrotoxicity, myelosuppression), and variable individual responses, which can lead to adverse effects and suboptimal long‐term outcomes in some cases [43, 44]. To date, Belimumab remains the only biologic approved by the FDA for adjunctive therapy in SLE. Clinical trials and real‐world evidence indicate that Belimumab leads to clinically meaningful improvement (e.g., reduced disease activity scores) in over 50% of patients, although rates of complete remission are lower [45, 46]. This underscores the potential of targeted biologics, yet highlights the ongoing need for more precise and sustained therapeutic strategies. Immunomodulators, such as therapeutic antibodies or immunometabolic inhibitors, necessitate high doses to attain therapeutic efficacy, often leading to substantial off‐target side effects [44]. Concurrently, certain chemical drugs are confronted with challenges such as inadequate solubility, a short plasma half‐life, and detrimental side effects [47]. Furthermore, in the context of SLE with Lupus Nephritis (SLE/LN), the intricacy of the autoimmune network necessitates the urgent development of novel strategies that target more selective cellular pathways, thereby enhancing efficacy while minimizing toxicity.

## 4 Application of Nanomaterials in the Treatment of SLE/LN

Nanomedicine represents an emerging and promising field in the realm of disease diagnosis and treatment, leveraging the advancements of nanotechnology. Nanotechnology offers a novel approach that holds considerable promise in disease diagnosis, treatment, and targeted drug delivery systems. Specifically, nanomaterials serve as carriers or vectors, facilitating the delivery of bioactive molecules to their primary targets with precision and efficiency. Nanomaterials can synergize to enhance the efficacy of the drug payload [8, 9]. Broadly speaking, nanomaterials are defined as materials with sizes spanning approximately from 10 to 1000 nanometers, encompassing a diverse spectrum of organic, inorganic, and polymeric nanostructures. These include, but are not limited to, nanoparticles, hydrogels, liposomes, micelles, dendritic polymers, mesoporous materials, adenoviruses, lysozyme, elastin‐like peptides, and chitosan. The size of nanoparticles significantly influences their biological activity, including their interactions with the reticuloendothelial system (RES), adjuvant characteristics, and susceptibility to phagocytosis [48, 49]. Nanoparticles enable more efficient and targeted delivery of chemotherapy drugs [50]. The utilization of nanoparticles can augment the solubility of poorly water‐soluble drugs, thereby facilitating drug delivery, mitigating drug resistance, enabling site‐specific drug targeting, enhancing the effective cytoplasmic penetration of drugs across epithelial and endothelial cell barriers, allowing for the codelivery of two or more drugs, and permitting the tracking of drug delivery sites [51].

**FIGURE 2:**
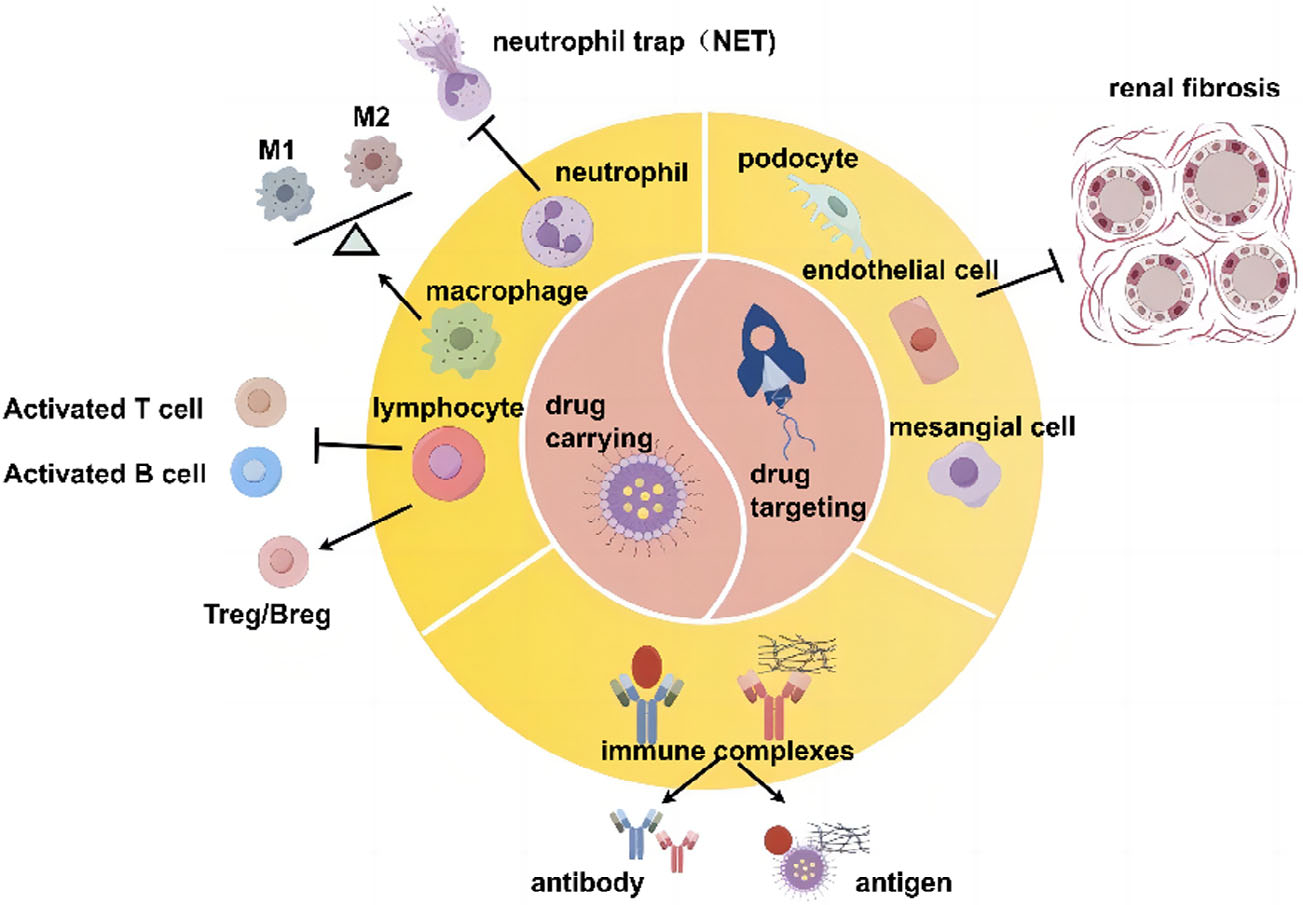
The dual purposes of nanomaterials in SLE/LN. (1) They can serve as carriers for biologics and small molecule drugs, facilitating their delivery; (2) They can enhance the targeting efficiency of drugs toward specific cellular targets, including immune cells, intrinsic renal tissue cells, and ICs.

Over the past decade, the development and application of nanotechnology as a carrier system has established a robust paradigm. Nanomaterials have been extensively investigated for the treatment of numerous diseases, including autoimmune disorders such as SLE and lupus nephritis [12, 13]. Nanomaterials are currently employed in applications targeting SLE and lupus nephritis (Figure 2), serving dual purposes: (1) they can be used as carriers for biologics and small molecule drugs, and (2) they enhance the targeting efficiency of drugs [52]. In the context of the immune system, nanomaterials currently utilized in nanomedicine can be internalized by cells of the reticuloendothelial system, which encompasses macrophages, dendritic cells (DCs), and neutrophils [48, 49]. Furthermore, these nanomaterials can undergo surface modification to specifically target lymphocyte subpopulations, such as T cells and B cells. In the field of renal disease, nanocarrier materials can facilitate more precise drug delivery and enhance therapeutic efficacy through some mechanisms, including active targeting, passive targeting, and other advanced strategies [10]. The nano‐drug delivery system, with specifically targeted CD4+ and CD8+ T cells, exhibits enhanced efficacy in inhibiting T cell activation, balancing the populations of effector T cells and Treg cells, and alleviating the hyperactivation of T lymphocytes in both SLE and lupus nephritis (LN) [53, 54]. The proliferation and activation of B cells are closely intertwined with the progression of LN. And by targeting B cells, the production of autoreactive antibodies can be significantly reduced, potentially leading to an amelioration of disease manifestations [55, 56]. The development of chronic inflammation in SLE is influenced by the impaired phagocytosis capability of apoptotic cells. By targeting renal macrophages, their phagocytosis can be restored and their anti‐inflammatory (M2 macrophage) transformation promoted [57]. Dendritic cells (DCs), as innate immune cells that function as antigen‐presenting cells (APCs), play a pivotal role in immune activation and immune tolerance. The in situ targeting of DCs with nanoparticles (NPs) may augment DC tolerance, thereby modulating the immune response [58, 59]. Neutrophil extracellular traps (NETs) have the capacity to activate and induce damage to vascular endothelial cells, a mechanism that can intensify kidney inflammation and contribute to the progression of small vessel lesions in individuals diagnosed with lupus nephritis. Nanomaterials targeting neutrophils can reduce NET production in neutrophils [60]. In recent nanomedical research endeavors focusing on SLE and lupus nephritis (LN), nano‐drug delivery systems have exhibited the capacity to not only specifically target the immune system but also to tackle the formation and deposition of ICs within both systemic circulation and renal tissue. Additionally, these advanced systems can be precisely targeted to mitigate excessive renal fibrosis, thereby providing a multitude of therapeutic advantages [10, 61, 62].

The interaction between nanocarriers and target cells is governed by specific mechanisms. Receptor‐mediated endocytosis is a primary pathway; for example, scavenger receptors (e.g., SR‐A) on macrophages facilitate uptake of anionic or ligand‐decorated nanoparticles, while Fc*γ* receptors (Fc*γ*R) on B cells can engage antibody‐conjugated nanocarriers [52, 55]. Lymphoid organ trafficking is crucial for modulating adaptive immunity. Nanoparticles sized 10–100 nm, especially with surface modifications (e.g., targeting ligands for lymphatic endothelial cells), can drain to lymph nodes, directly accessing T cells, B cells, and dendritic cells [63]. Furthermore, some nanomaterials can intrinsically modulate immune signaling pathways, such as Toll‐like receptor (TLR) pathways, by presenting pathogen‐associated molecular patterns (PAMPs), offering opportunities for designing combined delivery and immunomodulatory agents [64].

## 5 Advantages of Nanomaterials in the Treatment of SLE/LN

Traditional therapeutic agents are characterized by their suboptimal absorption profiles, limited solubility, and insufficient accumulation in target organs. Nanomaterial‐based drug delivery systems address these deficiencies of conventional medications, enabling precise drug targeting and sustained release capabilities [65, 66]. Furthermore, in contrast to nontargeted systemic administration, nanomaterial‐mediated targeted therapy facilitates the delivery of drugs to specific sites of interest, thereby enhancing therapeutic efficacy, allowing for dosage adjustments, and minimizing systemic toxicity. Nanomaterials exhibit a propensity to accumulate within inflammatory tissues, either through vascular leakage or via macrophage‐mediated processes. And they can be actively targeted through the binding to specific receptors or ligands [67]. The advantages of nanomaterials over traditional drugs are as follows: (1) By targeting specific cells and enhancing local concentrations upon release, the delivery of biologics can be significantly reduced by a factor of 100–1000 times, reducing side effects and costs; (2) The delivery of insoluble drugs can be enhanced to optimize their bioavailability; (3) The integration of a therapeutic agent with a diagnostic compound can be successfully achieved, yielding a “theranostic” drug. The ability to assemble these materials at the nanoscale facilitates the optimization of the drug's biological distribution, ensures specific interaction with extracellular receptors (when accurately targeted), and promotes efficient intracellular accumulation, all while preserving normal physiological functions [52]. Furthermore, nanomaterials exhibit the potential to amplify the cytoplasmic efficacy of drugs, thereby facilitating the concurrent delivery of two or more therapeutic agents and providing advantages such as the precise tracking of drug delivery locations [51, 68]. As the field of nanotechnology continues to advance, the employment of nanocarriers is poised to transform the paradigm of immunotherapy for SLE and LN, heralding a new era in the treatment of these conditions. Nanocarriers demonstrate distinct therapeutic benefits in tackling the complexities associated with systemic drug delivery. They enable highly precise targeting of specific organs or cells, while concurrently mitigating adverse reactions in a highly efficient manner.

Glucocorticoids and immunosuppressants have exhibited substantial efficacy in managing disease progression and diminishing the morbidity rates associated with SLE. However, these conventional chemical agents exhibit numerous drawbacks, such as limited solubility, a brief elimination half‐life, nonspecific tissue distribution, and intrinsic toxicity. These characteristics frequently result in adverse side effects and diminished patient adherence. Furthermore, the present therapeutic indicators for SLE medications, which are restrictive in terms of dosing, impede their optimal application. As a result, SLE patients often suffer from severe adverse effects arising from nonspecific organ toxicity, a direct consequence of frequent and prolonged treatment regimens. When compared to traditional therapeutic methodologies, nanotechnology‐based approaches exhibit a multitude of advantages. These include heightened stability, advantageous biodistribution profiles, extended drug release kinetics, diminished immunotoxicity, augmented drug resilience, and the high capacity for targeted drug delivery specifically to inflammatory tissues [13, 58, 68] . One of the most potent strategies for prolonging the cyclic administration of therapeutic agents entails encapsulating drugs within nanocarriers to formulate nanomedical preparations, holding paramount significance in the treatment of autoimmune diseases [69, 70, 71]. In SLE, nanoparticles loaded with nonspecific immunosuppressants or glucocorticoid prodrugs can enhance the pharmacokinetic and biological distribution profiles of the drug, while simultaneously mitigating its adverse effects [72, 73]. Furthermore, nanoparticles have also found application in the diagnosis of SLE. Nano‐delivery systems also exhibit a robust capability in efficiently transferring miRNAs, siRNAs, cytokines, and other biomolecules to targeted cells. Moreover, when compared to conventional drugs, these nanoparticles exhibit a preferential transfection efficiency specifically with activated immune cells [74].

The codelivery of multiple therapeutic agents via a single nanocarrier represents a powerful strategy to address the multifactorial pathogenesis of SLE/LN. Nanocarriers can encapsulate drugs with different mechanisms of action and ensure their synchronized delivery to the same target site. Key combination strategies include: (1) Immunosuppressants with Antioxidants: Coencapsulating drugs like rapamycin (immunosuppressant) with N‐acetylcysteine (antioxidant) could simultaneously suppress immune activation and mitigate oxidative stress, showing synergistic effects in lupus models [75, 76]. (2) Gene Therapy with Conventional Drugs: Combining siRNA or miRNA targeting key inflammatory cytokines (e.g., TNF‐*α*, IL‐6) with corticosteroids (e.g., dexamethasone) enables multilevel blockade of inflammation, potentially enhancing efficacy and reducing drug resistance [77]. (3) Immunomodulation with Anti‐fibrotic Therapy: For LN, nanocarriers targeting renal cells can codeliver anti‐inflammatory agents and anti‐fibrotic drugs (e.g., TGF‐*β* inhibitors), aiming to concurrently halt inflammatory processes and prevent chronic fibrosis [78, 79].

## 6 Diverse Nano‐Material‐Based Drugs in the Application of SLE/LN

**FIGURE 3:**
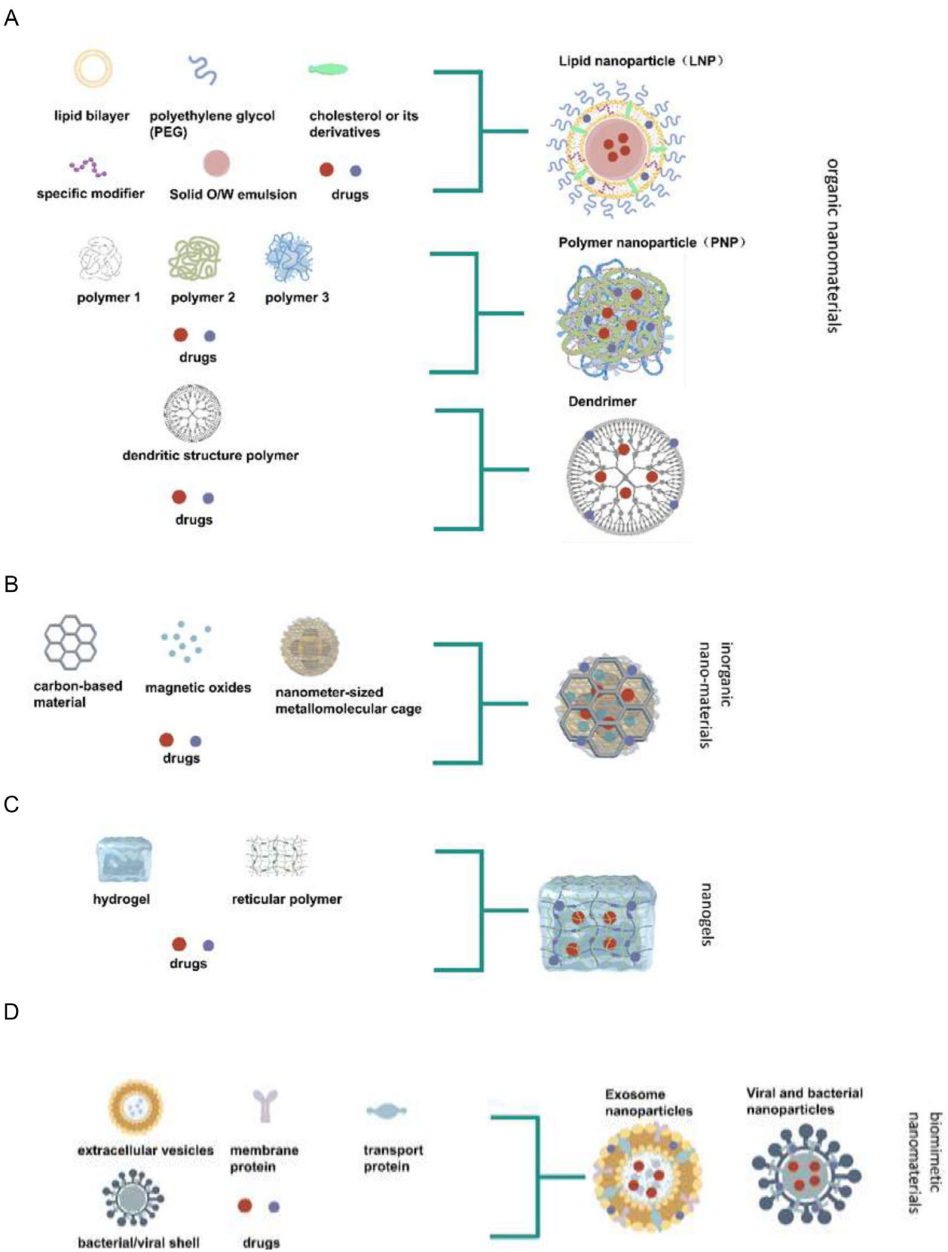
Application of nano‐drugs with different materials in SLE/LN. (A) organic nanomaterials; (B) inorganic nanomaterials; (C) nanogel; (D) Biomimetic nanomaterials.

Nanomaterials applied in SLE/LN therapy can be systematically classified into three fundamental categories: organic nanomaterials, inorganic nanomaterials, and biomimetic nanomaterials (Table 1 and Figure 3). This section synthesizes the key design principles and SLE/LN‐specific applications of each category, moving from classical to emerging platforms.

**TABLE 1:** The diverse nano‐material‐based drugs in the application of SLE/LN.

Type of nanomaterialss	Nanomaterials (NPs)	Drug delivery	Significance	Ref.
Lipid Nanoparticles (LNP)	cationic liposomes	Dihydroartemisinin and HMGB1‐siRNAs	Suppressed the proliferation and activation of B cells through the TLR4 signaling pathway, alleviated the progression of lupus nephritis	Diao et al. [80].
	Liposome	methylprednisolone hemisuccinate (MPS)	Suppressed anti‐dsDNA antibody levels, proliferation of lymphoid tissue and renal damage, and in prolonging survival of LN animal	Moallem et al. [81].
	apolipoprotein E3‐reconstituted high‐density lipoprotein (ApoE‐rHDL)	prednisolone disodium phosphate	Reduced the levels of inflammatory cytokines in the macrophages in vitro and alleviated lupus nephritis in MRL/lpr mice	Yu et al. [82].
Polymer Nanoparticles (PNP)	Nile Red‐labeled polylactic acid nanoparticles (NR‐PLA NPs)	JAK inhibitor baricitinib (BARI)	Dampened B‐cell activation, proliferation and plasma cell differentiation, inhibited key cytokine production	Álvarez et al. [83].
	a poly (ethylene glycol) (PEG)	dexamethasone (Dex) prodrug (ZSJ‐0228)	Reduced the proteinuria, improved renal histological scores and survival data in lupus‐prone NZB/W F1 mice	Zhao et al. [84].
	Poly (lactic‐co‐glycolic acid) (PLGA)	IL‐2 and TGF‐*β*	Enabled the expansion of Treg cells in vivo and inhibits pathogenic immune responses in SLE	Horwitz et al. [85].
	PHBVHHx	azathioprine (AZA)	Exhibited superior therapeutic efficacy to AZA and AZA‐polylactic acid (PLA) nanoparticles without appreciable toxicity	Hu et al. [86].
	vesicular “nanogel” platform	mycophenolic acid (MPA)	Extended the survival of lupus‐prone NZB/W F1 mice, was uptaked by dendritic cells, which impacts the quality of therapeutic immunosuppression	Look et al. [59].
Dendrimer	glycodendrimers (PAMAM) and (PPI)	—	Inhibited the formation of immunocomplexes	Tassinari et al. [61].
Inorganic nanomaterials	gold nanocage (AuNC)	liver X receptor (LXR) agonist T0901317	Enhanced apoptotic cell clearance, decreased production of anti‐dsDNA autoantibodies, reduced inflammatory response, and alleviated of kidney damage in lupus model mice	Xu et al. [57].
	superparamagnetic particles of iron oxide (SPIO)	—	Linked to the iC3b/C3d binding region of complement receptor type 2 (CR2) for monitoring disease activity in patients with lupus nephritis	Hasebroock et al. [87].
	a polydopamine (PDA)‐based nanocarrier modified with Fe3O4 and Pt nanoparticles	necrostatin‐1 (Nec‐1)	Combined these advantages exhibited outstanding performance in LN imaging and therapy, and offered valuable insights into LN diagnosis and therapy	Li et al. [88].
Nanogels	*β* or *γ*‐CD/PAA nanogels	dexamethasone	Combined faster action with lower doses, suggested the potential for being more manageable than the free drug, reducing its adverse side effects	Argenziano et al. [89].
	lipid bilayer cyclodextrins nanogels	Mycophenolic acid (MPA)	Effectively treated murine lupus, extended the mean survival time, reduced production of inflammatory cytokines such as IFN‐*γ* and IL‐12	Look et al. [90].
	lipid bilayer cyclodextrins nanogels	Inhibitor of CaMK4 KN93	Effectively blocked Th17 cell differentiation and expansion as measured in the spinal cords and kidneys of mice developing experimental autoimmune encephalomyelitis or lupus	Otomo et al. [91].
	cationic agarose hydrogel	TNF‐*α* antisense oligonucleotides (ASO)	Reduced TNF‐*α* expression in CD169(+) macrophages and inhibited lymphocytes over‐proliferation, resulted in the relief of the lupus‐like symptoms of the animals	Huang et al. [92].
Biomimetic nanomaterials	MSC‐ Exosome	dexamethasone	Increased the anti‐inflammatory inhibitory effect of CD4+T cells through the release of dexamethasone liposomes or Dex‐integrated MSC‐derived exosomes (Dex‐MSC‐EXOs)	Ma et al. [93].
	MSC‐ Exosome	miR‐146a‐5p	Diminished NOTCH1 expression to accelerate M2 macrophage polarization via delivery of miR‐146a‐5p, alleviated SLE‐associated DAH in mice	Chen et al. [94].
	EL‐4‐derived Exosome	curcumin	Enhanced anti‐inflammatory activity of curcumin without significant side effects due to innocent bystander or off‐target effects	Sun et al. [95].

### 6.1 Organic Nanomaterials

#### 6.1.1 Lipid Nanoparticles (LNP)

Lipid‐based systems, including liposomes, are versatile carriers capable of encapsulating both hydrophilic and hydrophobic agents. In SLE/LN, their surface properties are often modified to achieve active targeting or enhanced stability. A prominent example is the use of cationic liposomes for codelivery. Lu Diao et al. constructed TAT peptide‐modified cationic liposomes to deliver HMGB1‐siRNA and dihydroartemisinin (DHA), which synergistically suppressed B‐cell proliferation and alleviated lupus nephritis in mice via the TLR4 pathway [80]. Beyond nucleic acids, liposomes effectively deliver steroids. Moallem E. et al. developed a liposomal formulation of methylprednisolone hemisuccinate (MPS) that demonstrated superior accumulation in inflammatory tissues and stronger therapeutic efficacy in MRL/lpr mice compared to free MPS [81]. Another innovative approach utilized biomimetic lipid nanoparticles. Yu Y. et al. engineered a steroid‐loaded nanocarrier based on apolipoprotein E3‐reconstituted high‐density lipoprotein (ApoE‐rHDL). This PLP‐CaP‐rHDL system efficiently targeted macrophages in vitro and delivered prednisolone phosphate to inflammatory sites in lupus‐prone mice, reducing cytokine levels without significant side effects [82].

#### 6.1.2 Polymer Nanoparticles (PNP)

Synthetic biodegradable polymers like poly(lactic acid) (PLA) and poly(lactic‐co‐glycolic acid) (PLGA) are FDA‐approved materials favored for their controlled release profiles. Their functionality is often enhanced through surface engineering. For instance, PEGylation—the conjugation of polyethylene glycol—is a common strategy to improve solubility, prolong circulation, and reduce immunogenicity.Alvarez K's study revealed that PLA NPs encapsulating AK1 and JAK2 kinase inhibitors exhibit therapeutic potential for targeting B‐cell disorders, including SLE/LN [83]. Zhao Z. et al. developed a PEG‐based dexamethasone prodrug nanoparticle (ZSI‐0228) that altered the drug's pharmacokinetics to favor renal distribution, thereby improving LN pathology while mitigating systemic glucocorticoid toxicity in NZB/W F1 mice [84]. Polymers can also be designed for cell‐specific targeting. Álvarez K. et al. used PLA nanoparticles to encapsulate the JAK1/2 inhibitor baricitinib (BARI). These Nile Red‐labeled PLA NPs were selectively internalized by CD19+ B cells, dampening their activation, plasma cell differentiation, and pathogenic cytokine production, highlighting a targeted strategy for B‐cell‐driven pathology [80]. Furthermore, polymers serve as platforms for cytokine delivery to modulate immune responses. Horwitz D.A. et al. employed PLGA nanoparticles loaded with IL‐2 and TGF‐*β* to expand regulatory T cells (Tregs) in vivo, which suppressed pathogenic immune responses in a lupus model [85]. Other biocompatible polymers like polyhydroxyalkanoates (e.g., PHBVHHx) have also been explored, showing enhanced delivery of azathioprine for SLE treatment [86].

#### 6.1.3 Dendrimer

Dendrimers are highly branched, monodisperse macromolecules with a well‐defined structure and multivalent surface functionalities. This unique architecture allows them to interact with multiple biomolecules simultaneously. In SLE, a critical pathogenic event is the formation of ICs between anti‐dsDNA antibodies and DNA. Cationic dendrimers, such as polyamidoamine (PAMAM) and polypropylenimine (PPI) types, exploit electrostatic interactions. Their positively charged terminal amine groups under physiological conditions can bind to negatively charged DNA and autoantibodies, thereby inhibiting the formation or promoting the dissolution of these pathogenic ICs. This mechanism offers a direct, nanomaterial‐based intervention upstream of inflammation and tissue deposition, as demonstrated in recent studies [61, 96].

### 6.2 Inorganic Nanomaterials

Inorganic nanoparticles, including metals and metal oxides, offer distinct properties such as superparamagnetism for imaging or plasmonic effects for therapy. Their application in SLE/LN often combines these properties with targeted drug delivery. Gold‐based nanoparticles have been engineered for specific targeting. Xu N. et al. designed a gold nanocage (AuNC) system coated with a lipid bilayer and conjugated with phosphatidylserine (PS) to mimic apoptotic cells. This “camouflaged” nanocage delivered a liver X receptor (LXR) agonist (T0901317) to macrophages, enhancing the clearance of apoptotic debris—a key defective process in SLE—and reducing autoantibody production and kidney injury [49, 57]. Superparamagnetic iron oxide nanoparticles (SPIONs) are invaluable for diagnosis and monitoring. They can be functionalized with targeting ligands to visualize disease activity. For example, SPIONs attached to a complement receptor (CR2) ligand were used to detect tissue‐bound complement fragments (iC3b/C3d), providing a noninvasive method to monitor LN activity in mice [87]. Moving toward theranostics, Li M. et al. developed a polydopamine‐based nanoparticle codecorated with Fe_3_O_4_ and Pt. This platform, loaded with a RIPK1 inhibitor, enabled simultaneous fluorescence/MRI bimodal imaging and therapy in a LN model, showcasing the potential for integrated diagnosis and treatment [88].

### 6.3 Nanogels

Nanogels, or hydrogel nanoparticles, merge the high water content and biocompatibility of hydrogels with the nanoscale size required for drug delivery. Their cross‐linked network allows for high drug loading and stimuli‐responsive release. In SLE/LN, they have been tailored for sustained and targeted drug release. Argenziano M. et al. developed a cyclodextrin‐based nanogel for dexamethasone delivery, which aimed to enhance drug solubility and prolong its contact with inflamed tissues [89]. A particularly effective design involves a core–shell structure. Look M. et al. created a vesicular nanogel featuring an internal aqueous PEG‐PLA core (for solubilizing hydrophobic drugs like mycophenolic acid, MPA) surrounded by an outer lipid bilayer. This hybrid construct showed greater drug loading than traditional liposomes and was more efficiently internalized by dendritic cells, leading to improved survival and reduced renal damage in lupus‐prone mice [59, 90]. Nanogels also facilitate cell‐specific oligonucleotide delivery. Otomo K et al. demonstrated that a CaMK4 inhibitor (KN‐93) can be delivered to CD4+T cells via nanogel at a tenth of the standard drug dose, achieving the same clinical effect [91]. Huang Z. et al. used a cationic agarose nanogel to deliver TNF‐*α* antisense oligonucleotides (ASO) specifically to CD169+ macrophages in the spleen, effectively reducing TNF‐*α* expression and ameliorating lupus‐like symptoms in MRL/lpr mice [92].

### 6.4 Biomimetic Nanomaterials

#### 6.4.1 Exosome

Exosomes are endogenous, cell‐derived nanovesicles that play key roles in intercellular communication. Their natural composition makes them ideal, low‐immunogenicity carriers. In SLE/LN, mesenchymal stem cell (MSC)‐derived exosomes have shown therapeutic promise, either as innate immunomodulators or as engineered delivery vehicles. Ma W. et al. loaded dexamethasone into MSC‐derived exosomes (Dex‐MSC‐Exos), which synergistically enhanced the anti‐inflammatory function of CD4+ T cells and mitigated disease progression in lupus mice [93]. Furthermore, exosomes can deliver regulatory genetic material. Chen X. et al. demonstrated that exosomes from human umbilical cord MSCs transferred miR‐146a‐5p to M1 macrophages, promoting their polarization to the anti‐inflammatory M2 phenotype by downregulating NOTCH1, thereby alleviating inflammation [94]. Sun D et al. showed that exosome encapsulated curcumin enhanced its therapeutic power, effectively reduced macrophage activation and macrophage B cell activator (BAFF) secretion, effectively reduced kidney inflammation [95]. To overcome limitations in natural exosome production and drug loading, engineered artificial exosomes are being developed, offering more controllable and scalable platforms [97].

#### 6.4.2 Biomimetic Nanomaterials Based on Cell Membranes

This strategy involves coating synthetic nanoparticle cores with natural cell membranes (e.g., from immune cells, red blood cells), endowing the nanoparticles with the surface antigens and functions of the source cell. This “camouflage” can evade immune clearance and confer active targeting. For instance, nanoparticles coated with T regulatory (Treg) cell membranes, which retain surface proteins like CTLA‐4, have been designed to engage with APCs and effector T cells, mimicking Treg's immunosuppressive contact [98]. Liu et al. developed sLipo leva, a stem cell bionic liposome drug delivery system formed by fuzing drug‐encapsulated liposomes with a dry cell membrane, targeting macrophages and activating T cells [99]. Similarly, macrophage membrane‐coated nanoparticles can inherit the ability to target inflammatory sites [100]. This approach represents a powerful fusion of synthetic nanomaterials with complex biological interfaces.

#### 6.4.3 Biomimetic Nanomaterials Based on Viruses and Bacteria

Virus‐like particles (VLPs), derived from viral capsid proteins, and nanomaterials based on bacterial membranes are explored for their inherent immunomodulatory properties, primarily in vaccine development. Their highly organized, repetitive structures are potent activators of the immune system, which can be harnessed to induce tolerance or immunity [11, 101, 102]. While their direct application in SLE/LN therapy is still emerging, they represent a promising frontier for developing tolerogenic vaccines or targeted immunomodulators, leveraging pathogen‐mimetic structures to steer the immune response in a desired direction.

## 7 Application of Different Targeted Nanomedicine in SLE/LN

**FIGURE 4:**
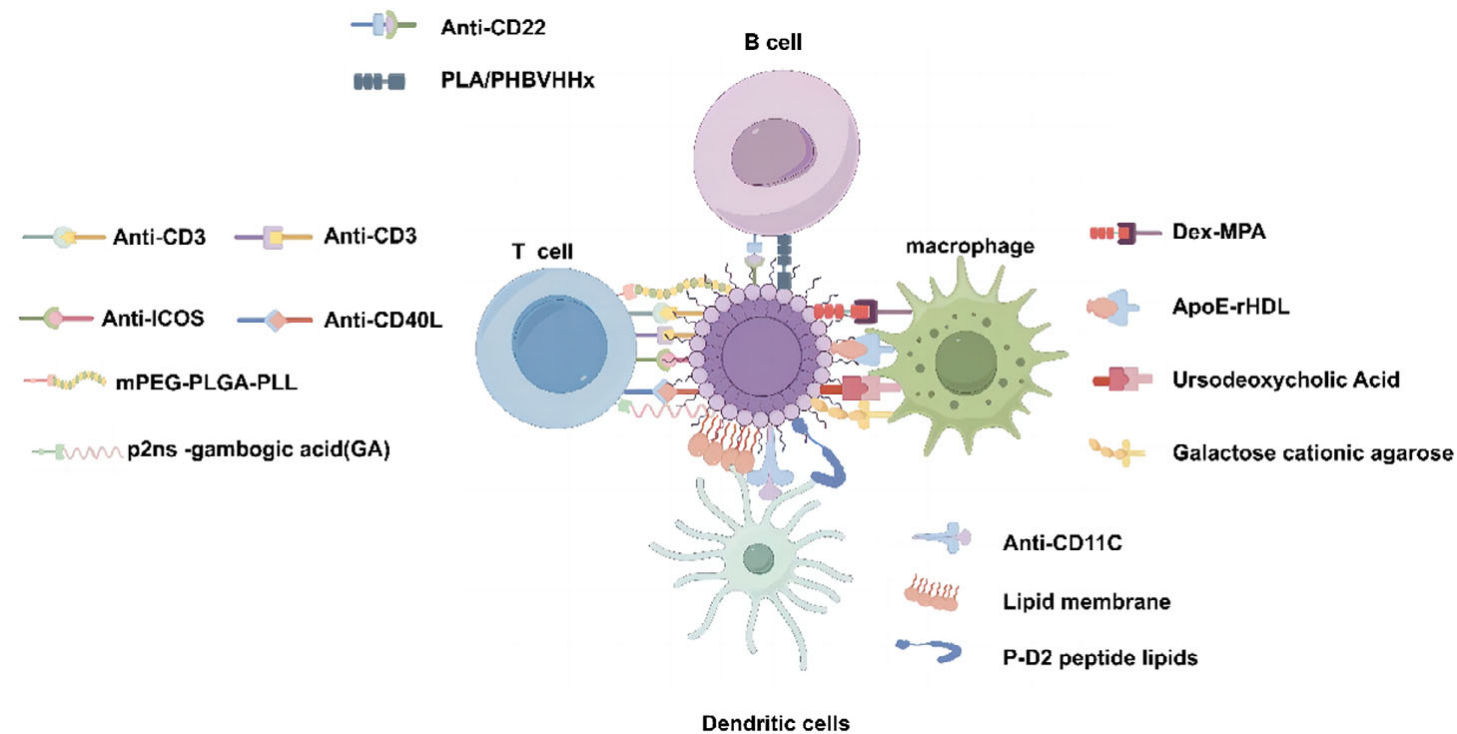
Application of nanomedicine targeting immune cells in SLE/LN. Selective targeting and delivery of therapeutic agents to autoreactive immune cells, encompassing T cells, B cells, dendritic cells, and macrophages, is achieved through precise ligand‐receptor binding interactions, thereby facilitating the modulation of an overreactive immune system.

**FIGURE 5:**
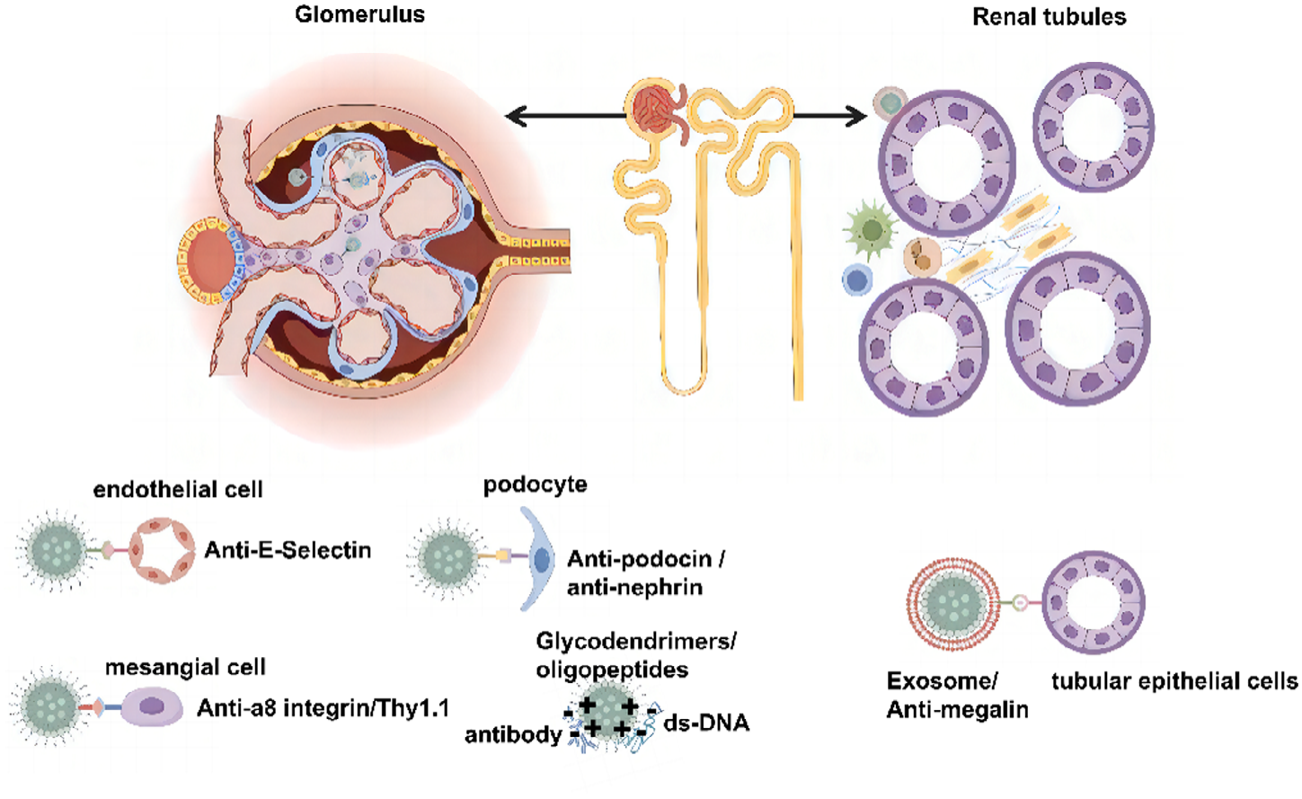
Application of nanomedicine targeting renal intrinsic cells and ICs in SLE/LN. Selectively deliver drugs to ds‐DNA or antigens to inhibit the formation of ICs; selectively target drug delivery to renal resident cells, including mesangial cells, podocytes, and epithelial cells, to repair kidney injury.

Nanoparticles are designed for targeted cell/tissue delivery with high drug‐loading capacity, improving drug delivery and representing a new generation of biomedical drug delivery systems. The optimal drug delivery system for SLE/LN is to deliver the drug to abnormally responsive immune cells and intrinsic cells at the site of renal lesions, while the drug carrier degrades metabolism without accumulation in the normal physiological environment. Nanoparticle‐based drug delivery systems can be used to treat SLE/LN (Table 2): reduce an overactive immune response by selectively target autoreactive immune cells (T, B, dendritic, macrophages) (Figure 4); inhibit IC formation by selectively targeting ds‐DNA; and repair kidney damage by selectively delivering drugs to mesangial, podocytes, and epithelial cells (Figure 5). Nanomaterial‐based targeted delivery systems are an ideal approach for future drug and signaling molecular inhibitor evaluation.

**TABLE 2:** The diverse targeted nanomedicine in the application of SLE/LN.

Type of targets	Decoration	Nanomaterials	Drug delivery	Significance	Ref.
Targeting T cells	anti‐CD3/CD28	PLGA	IL‐2 and TGF‐*β*	Induced both CD4+ and CD8+ T cells to become FoxP3+ T regulatory cells (Tregs), sustained Treg activity with prolonged efficacy in humanized mice	Giang et al. [54].
	anti‐CD4	nanogels	mycophenolic acid (MPA)	Decreased IFN‐*γ*+ CD4 T cells, reduced production of inflammatory cytokines such as IFN‐*γ* and IL‐12	Look et al. [90].
	MHC target antigens	PLGA	regulatory molecules	Increased regulatory T cells, inhibited T cell proliferation and elevated T cell apoptosis in spleen	Pei et al. [103].
	gambogic acid (GA)	P2Ns	Cyclosporine A (CsA)	Enhanced the targeting of lymphatic tissues, transforming CsA into a potent single therapeutic for SLE by GA association with CD71+ lymphocytes	Ganugula et al. [104].
	—	mPEG‐PLGA‐PLL	MicroRNA‐125a	Delivered miR‐125a into splenic T cells, exhibited good biocompatibility and protected miR‐125a from degradation, prolonged the circulatory time of miRNA, versed the imbalance of effector/regulatory T cells	Zhang et al. [74].
Targeting T cells and B cells	anti‐ICOS and anti‐CD40L	PEG	rapamycin (RAP)	Selectively targeted SLE Th cells and potently inhibited Th–B‐cell reciprocal activation by targeting dual costimulatory pathways	Zhang et al. [53].
Targeting B cells	CD22 ligand	liposomes	STALs	Induced a tolerogenic program that selectively causes apoptosis in mouse and human B cells	Macauley et al. [55].
	—	NR‐PLA	JAK inhibitor baricitinib (BARI)	Internalized by CD19+ B cells, dampened B‐cell activation, proliferation and plasma cell differentiation, inhibited key cytokine production	Álvarez et al. [83].
	—	PHBVHHx	azathioprine (AZA)	Increased uptake and intracellular internalization by B cells, exhibited superior therapeutic efficacy to AZA and AZA‐polylactic acid (PLA) nanoparticles without appreciable toxicity	Hu et al. [86].
Targeting dendritic cells	ApoB‐100 derived antigenic peptide P210	PEG‐b‐PPS	1, 25‐Dihydroxyvitamin D3 (aVD)	Blinded CD11c on the DC surface, enhanced the cytosolic delivery and resulting immunomodulatory capacity of aVD of kidney damage in lupus model mice	Yi et al. [105].
	—	Nanogels	mycophenolic acid (MPA)	Nanogels were more effectively internalized by dendritic cells, extended the survival of lupus‐prone NZB/W F1 mice	Look et al. [59].
Targeting macrophages	ApoE‐rHDL	lipidosome	prednisolone disodium phosphate (PLP)	Reduced the levels of inflammatory cytokines in the macrophages in vitro and also effectively alleviated lupus nephritis in MRL/lpr mice without causing obvious side effects	Yu et al. [82].
	ursodeoxycholic acid (pUDCA)	rapamycinNP polymerization	rapamycin (RAP)	NPs was absorbed by monocytes, and intestinal macrophages with high expression of bile acid TGR5 receptor and promoted differentiation of macrophages into M2 anti‐inflammatory macrophages adverse side effects	Lee et al. [106].
Targeting macrophages	phosphatidylserine (PS)	gold nanocage (AuNC)	liver X receptor (LXR) agonist T0901317	PS‐lipos‐AuNC@T0901317 could efficiently enhance apoptotic cell clearance by elevating the expression of Mer of macrophages, resulting in decreased production of anti‐dsDNA autoantibodies, reduced inflammatory response in lupus model mice	Xu et al. [57].
	—	dextran mycophenolate‐based nanoparticles	mycophenolic acid (MPA)	MPA@Dex‐MPA NPs in the spleen and kidney, where they were mostly phagocytosed by macrophages, made macrophages be polarized toward a CD206+ M2‐like phenotype, with a downregulation of surface CD80 and CD40, and reduced TNF‐*α* production in the spleen and kidney	Otomo et al. [91].
	—	galactose cationic agarose	TNF‐*α* antisense oligonucleotides	Reduced TNF‐*α* expression in CD169(+) macrophages and inhibited lymphocytes over‐proliferation, resulted in the relief of the lupus‐like symptoms of the animals	Huang et al. [92].
Targeting macrophages	—	HUCMSC‐EXOs	miR‐146a‐5p	HUCMSC‐derived exosomes diminished NOTCH1 expression to accelerate M2 macrophage polarization via delivery of miR‐146a‐5p, thus alleviating SLE	Chen et al. [94].
Targeting immune complex	open‐shell maltose	PAMAM and PPI dendrimers	—	Glycodendrimers had a better activity of glycodendrimers compared to oligopeptides, inhibited the formation of immunocomplexes	Tassinari et al. [61].
	D‐form modified ALW	PEG‐PLGA	—	Reduce the glomerular deposition of IgG and C3, improve renal histopathologies, such as glomerular proliferation and inflammatory cells infiltration, and markedly prolong lifespan in MRL/lpr lupus‐prone mice	Wang et al. [62].
	Anti‐IgD	dextran	—	Reduce IgG production in LPS‐stimulated normal animals, delayed the development of glomerulonephritis and improved survival	Shirai et al. [107].
	—	supermagnetic iron oxide particles	—	Detected tissue‐bound iC3b/C3d by attaching supermagnetic iron oxide particles (SPIO) to CR2's iC3b/C3d binding region and monitored disease activity in MRL/lpr mice	Hasebroock et al. [87]
Targeting renal podocytes	Anti‐ podocin or Anti‐nephrin	nanolipogels	CaMK4 inhibitor KN93	Podocyte‐specific delivery of a CaMK4 inhibitor prevented and reversed podocyte injury in lupus nephritis and podocytopathies	Maeda et al. [108].
Targeting renal mesangial cells	anti‐alpha8 integrin	Liposomes	—	Anti‐alpha8 integrin immunoliposomes were specific delivered to the mesangium following tail vein injection in mice	Scindia et al. [109].
	OX7 mAb	immunoliposomes	—	Deposited in the glomeruli and co‐located with mesangial cell markers	Tuffin et al. [110].
Targeting renal glomerular endothelial cells	anti‐E‐selectin	liposomes	dexamethasone	The Ab (Esel) liposomes colocalized with the endothelial cell marker CD31. Targeted delivery of dexamethasone by Ab (Esel) liposomes reduced glomerular endothelial activation.	Asgeirsdóttir et al. [111].
	—	Lyme‐Exos	steroid	Taken up by the kidney endothelial cell, downregulated related disase genes in the renal cortex and glomerular endothelial cells (GECs), affected the progression of Lupus nephritis	Chen et al. [112].
Targeting renal glomerular epithelial cells	—	MSC‐ Exos	miR‐let‐7c	miR‐let‐7c from MSCs was be delivered to mouse renal epithelial cells via exosomes	Wang et al. [113].
	megalin ligand‐ cilastatin	H‐Dot nanotherapeutics	dexamethasone	Kidney‐specific nanoparticle enabling specific within‐kidney targeting to proximal tubular epithelial cells provided by the megalin ligand cilastatin to improve the safety and efficacy of dexamethasone	Funahashi et al. [114].

### 7.1 Targeting T Cells

SLE is an autoimmune chronic inflammatory disease influenced by genetic, endocrine, environmental, and immune factors. Targeted therapy for different aspects of SLE pathogenesis is a new trend, and T cell targeted therapy is a specific therapy for autoimmune initiation [115, 116]. T cell immunotherapy for SLE/LN hinges crucially on therapeutic target selection, and improper target selection may lead to more serious consequences. Thus, research must identify appropriate therapeutic targets. Safe, effective approaches for targeted drug development must ensure T cell efficacy. Due to limitations in internalization mechanisms, T cells pose challenges for intracellular drug delivery compared to other immune cells. Nanomedicine can guide drugs’ uptake and endosome escape by enhancing targeted T cell via surface modification [101].

Nanomaterials coated with anti‐T cell surface antibodies or peptide/MHC complexes can function as artificial APCs, targeting CD4 and CD8 T cells for TCR stimulation. Giang S et al. studied aAPC NPs (PLGA‐based) modified with anti‐CD3/CD28 and loaded with IL‐2 and TGF‐b, enhancing in vitro and in vivo production and expansion of CD4+FoxP3+ and CD8+FoxP3+ Tregs. This targeted enhancement of IL‐2 and/or TGF‐b aimed to repair immune deficiencies in autoimmune diseases like SLE [54]. Look M et al. discovered that, compared to nontargeted preparations, a nano‐gel system with CD4 antibody effectively targeted T cell delivery of mycophenolic acid, alleviating glomerulonephritis and reducing IFN‐*γ*+CD4 T cells in LN mice more efficiently [90]. Zhang J's team utilized RAP‐encapsulated PEG‐DSPE liposomes as scaffolds to covalently anchor F(ab’)2 fragments of *α*‐ICOS and *α*‐CD40L on NP surfaces, constructing ICOS/CD40L bi‐specific RAP NPs [53]. Continuous release of RAP from COS/CD40L bi‐specific NPs with precise targeting ability inhibits Th and B cells in situ, prevents Th‐B cell interactions, and significantly alleviates SLE progression in both induced and spontaneous lupus models. Pei W et al. discovered that peptide/MHC complex‐coated NPs targeted Th1 T cells for conversion into Treg cells [103].

In addition, some special nanomaterials also have the function of targeting T cells. Zhang J et al. developed a nano‐delivery system composed of monomethoxy (polyethylene glycol)‐poly (D, L‐lactide‐co‐glycolide)‐poly(L‐lysine) (mPEG‐PLGA‐PLL) for the delivery of miR‐125a to splenic T cells [74]. The mPEG‐PLGA‐PLL (PEALmiR‐125a) nanoparticles (NPs) loaded with miR‐125a exhibited excellent biocompatibility and protected miR‐125a from degradation, thereby prolonging the circulation time of the miRNA in vivo. Furthermore, they significantly alleviated the progression of SLE by reversing the effector/regulatory T cell imbalance. In this nano‐delivery system, the positively charged PLL ligand aided T cell internalization. Ganugula R studied and synthesized biodegradable ligand‐conjugated nanoparticles [p2 ns‐gambogic acid (GA)] that target CD71. CD71 (transferrin receptor 1) was expressed in mature and precursor lymphocytes. The binding of GA‐CD71 likely occurred at the extracellular protease‐like domain of CD71, facilitating clathrin‐dependent, receptor‐mediated endocytosis of the nanoparticles. Utilizing this nano‐drug delivery system to encapsulate cyclosporine (CsA) enhances its targeting to lymphoid tissues, improved CsA efficacy, and reduces toxicity [104].

### 7.2 Targeting B Cells

The proliferation and activation of B cells are closely associated with the development of LN. B cells that produce autoantibodies play an additional pathogenic role in the progression of SLE by secreting inflammatory cytokines. The production of autoantibodies, particularly those targeting double‐stranded DNA (dsDNA) and RNA‐containing antigens, is a hallmark feature of SLE. Several therapeutic strategies currently target B cells, including B‐cell depletion via anti‐CD20 mAbs [117, 118], BAFF inhibition [119], CAR T‐cell therapy targeting B‐cell antigens (e.g., CD19) [120, 121], and inhibition of signaling molecules such as BTK and PI3K kinases involved in B‐cell activation [122, 123]. However, these compounds are associated with adverse reactions such as anemia, neutropenia, thrombocytopenia, and an increased risk of severe infections. Nanomaterials can deliver them to specific B‐cell populations, reducing drug dosage and enhancing local therapeutic effects, thereby mitigating related adverse reactions.

B cells can internalize numerous molecules through endocytosis mediated by BCR, TLRs, Fc receptors, and complement receptors (CRs) such as CD35 (CR1) and CD21 (CR2) [124]. Álvarez K designed a drug delivery system utilizing Nile Red (NR)‐labeled polylactic acid (PLA) nanoparticles (NPs) loaded with inhibitors of JAK1 and JAK2 kinases (BARI) [83]. This system targets B cells, significantly reducing their differentiation into plasma cells and the production of IgG/IgM and cytokines (IL‐10 and IL‐6), thereby improving the progression of LN. Polylactic acid (PLA) was a linear aliphatic polyester with lactic acid as its structural basis. The NR‐PLA NPs were designed to bind specifically to B cells, rather than other circulating leukocyte subpopulations. Furthermore, the physicochemical properties of NPs (size, PDI, charge, *ζ*‐potential) influence their interactions with various cell types, endocytosis pathways, and subsequent effects. Variations in particle size and *ζ*‐potential of PLA NPs may impact their interactions with B cells. The NR‐PLANPs constructed in this study had a size of about 140 nm and a *ζ* potential of 30 mV. Hu J et al. developed a novel drug delivery system using a biocompatible PHA terpolymer (PHBVHHx), loading immunosuppressant AZA onto PHBVHHx nanocarriers for the treatment of SLE [86]. Polyhydroxyalkanoates (PHAs) were noncytotoxic polyesters synthesized by bacteria in excess carbon and nitrogen conditions. T and B cells could efficiently uptake and internalize AZA‐PHA nanoparticles. Macauley MS et al. discovered that CD22‐ligand‐coated antigen nanoliposomes induce antigen‐specific B cell tolerance and apoptosis [55].

### 7.3 Targeting Dendritic Cells

Dendritic cells (DCs) are the most efficient APCs, constituting a pivotal linkage between innate and adaptive immunity. The development and pathogenesis of SLE are linked to abnormal DC homeostasis and function regulation. Thus, DC‐targeted therapy holds significant importance in treating SLE and autoimmune diseases. The high reactivity and tolerance changes of DC are related to the development and pathogenesis of autoimmune diseases. Consequently, targeted DC therapy via nanoparticle‐mediated drug delivery to induce auto‐tolerance has emerged as a promising research focus for autoimmune disease treatment [58, 59, 125]. Internalization of nanomaterials can be enhanced by ligands of CD11c, DEC‐205, other lectins, or TLRs [126, 127, 128]. Maximizing particle‐dendritic cell interactions is a critical consideration in nanomaterial design.

A biodegradable PLGA nanoparticle matrix is widely used in drug and antigen delivery, including targeting dendritic cells. Look M et al. discovered that lipid‐like vesicle “nanogels” were internalized more effectively by dendritic cells compared to solid biodegradable PLGA systems [59]. The nanogel comprised a lipid membrane with a 2:1 PC:cholesterol molar ratio and 3.2% pegylated DSPE, encapsulating a PEG‐PLA core and coated with hydrophobic MPA. These particles exhibited higher drug loading capacity than liposomes. Encapsulating MPA in nanogels improved survival rates and delays kidney damage in lupus‐prone NZB/WF1 mice.

Nanoparticle size influences DC targeting, with a range of 100–600 nm facilitating DC internalization and absorption [129]. Saengruengrit C et al. compared in vitro uptake of 300 nm and 500 nm PLGA NPs by DCs, finding higher uptake efficiency for 500 nm NPs [130]. Yi et al. demonstrated that CD11c on DC surfaces can be bound by density‐modified P‐D2 peptide lipid structure polymers, significantly enhancing cytoplasmic delivery and immunomodulation [105].

### 7.4 Targeting Macrophages

Inflammation is pivotal in SLE/LN pathogenesis, and its inhibition holds promise as a strategy. Macrophages are crucial components of the inflammatory cascade. Chronic inflammation in SLE enhances monocyte/macrophage inflammatory phenotype, altering their function, reducing phagocytic capacity, and exacerbating kidney damage in lupus nephritis. Dysregulation of M1/M2 in macrophages leads to abnormal clearance of apoptotic cells, which is another important mechanism in SLE/LN disease. Macrophage targeting is a crucial aspect of immune‐regulating drug delivery vectors. Nanomedicine can achieve systematic delivery or targeted accumulation in macrophage‐rich areas through passive and active targeting strategies [131]. Leveraging macrophage phagocytosis, NPs can encapsulate drugs to polarize these cells toward an anti‐inflammatory phenotype. These drugs encompass cytokines (e.g., IL‐10), statins, angiotensin receptor blockers, PPAR‐*γ* agonists, cytotoxic drugs, immunosuppressants, and others [132]. Macrophage surface receptors can further enhance nanomaterial targeting.

Jiang B et al. prepared glucan‐MPA conjugated NPs (Dex‐MPA NPs) via phacoemulsification and solvent evaporation [133]. These NPs were predominantly phagocytosed by macrophages in mouse spleen and kidney, promoting M2 differentiation and alleviating LN tissue damage. Getts et al. demonstrated that negatively charged PLGA NPs were absorbed by splenic macrophages expressing the clearance receptor MARCO [106]. Yu et al. developed a corticosteroid nanomedical delivery system using ApoE‐rHDL and CaP cores to load PLP, constructing PLP‐CaP‐rHDL for SLE treatment [82]. Recombinant high‐density lipoprotein (rHDL) is a typical nanoparticle less than 100 nm in diameter that accumulates at the site of inflammation through enhanced permeability and retention (EPR) effects. HDL could bind cellular receptors via apolipoproteins, such as scavger receptor Type BI (SR‐BI), ATP‐binding cassette transporter A1 (ABCA1) receptor, and ATP‐binding cassette transporter G1 (ABCG), which are abundant in macrophages. Thus, rHDL exhibits considerable potential in targeting drug delivery to inflammatory sites and macrophages. Lee et al. studied that pUDCA NPs containing rapamycin were absorbed by monocytes and intestinal macrophages expressing high levels of the bile acid TGR5 receptor, promoting their differentiation into M2 anti‐inflammatory macrophages. Huang et al. found that galactose cationic agarose transports TNF‐*α* antisense oligonucleotides to CD169+ splenic macrophages expressing galactose receptors, reducing TNF‐*α* expression, inhibiting lymphocyte proliferation, and alleviating lupus‐like symptoms [92].

Mimicking the macrophage phagocytosis of apoptotic cells, in addition to binding macrophage receptors, achieves macrophage targeting. The recognition of phosphatidylserine (a component of cell membranes) by macrophages serves as a potent apoptotic signal, enhancing the production of tolerogenic IL‐10 and TGF‐*β* [134]. Xu N et al. rationally designed and prepared an apoptotic cell‐mimicking gold nanocage (AuNC) nanodrug carrier by coupling phosphatidylserine (PS) to lipid‐coated AuNCs for the delivery of the liver X receptor (LXR) antagonist T0901317, which corrected impaired apoptotic cell clearance in SLE [57]. This nanosystem enhanced apoptotic cell clearance by upregulating Mer, a key phagocytic receptor on macrophages, thereby reducing anti‐dsDNA autoantibody production, alleviating inflammation, and mitigating kidney damage in LN. Exosomes primarily accumulate at the mononuclear phagocytic system (MPS)‐active sites upon intravenous administration. Artificial modification of exosome membranes to elevate target cell delivery is crucial for enhancing bioavailability. Artificially modified exosome membranes enhance drug delivery for targeted cell, crucial for improving drug bioavailability [135]. Chen X et al. discovered that exosomes from human umbilical cord mesenchymal stem cells transmit miR‐146a‐5p to M1 macrophages, promoting their polarization to M2 macrophages by downregulating NOTCH1 expression, thereby alleviating the inflammatory response in LN [94].

### 7.5 Targeting Immune Complex

A variety of autoantibodies, including anti‐ds‐DNA, anti‐SS‐DNA, anti‐RNA, and anti‐phospholipid, are observed in SLE. Notably, anti‐ds‐DNA antibodies are the primary pathological mediators in SLE. Anti‐ds‐DNA antibody, a serological marker of SLE, forms ICs with dsDNA in the blood, disseminating throughout the body and inducing symptoms like lupus glomerulonephritis. Under normal conditions, ICs form and are cleared. In SLE, however, their clearance is impaired, leading to the recruitment of inflammatory mediators and resulting in tissue damage and pathological manifestations. The pathological manifestations of the disease can be reduced by disrupting or suppressing the formation of ICs. From a therapeutic point of view, no cure has yet been found for this disease. Many recent studies have considered targeting SLE at a more “upstream” level, that is, focusing on the formation of ICs between antibody‐anti‐(ds)DNA and (ds)DNA. With this strategy in mind, various nanomaterials that could cause the IC to dissolve or prevent its formation were found. These nanoparticles primarily function through the following mechanisms: (i) they interact with double‐stranded DNA; (ii) they engage with antibodies; and (iii) they interact with ICs [61].

In this regard, dendritic macromolecules (dendrimers) exhibit a unique capability to interact with such nanomaterials. Dendrimers, a specific class of starburst and highly branched nano‐polymers, have garnered extensive attention within the scientific community due to their immense potential in the realm of biomedicine. Consequently, they have been widely utilized in various scientific endeavors. These nanomaterials use their own positive charge to interact with negatively charged DNA and antibodies to prevent the formation of ICs against ds‐DNA antibodies and ds‐DNA, or to destroy formed DNA. In several recently published papers on its possible applications in biomedicine, the third‐generation cationic polyaminamine (PAMAM) dendrimer has shown good activity in dissolving and preventing the formation of SLE associated ICs. Tassinari et al. synthesized multigeneration maltose‐modified PAMAM and PPI dendrimers and analyzed their anti‐SLE activities [61]. Both macromolecules inhibited IC formation and partially disrupted preformed ICs.

Based on the pathogenicity of anti‐ds‐DNA antibodies in SLE, therapeutic peptides targeting anti‐DNA antibodies, including ALW, hCDR1, pCONs, DWEYS, and file‐412, have been used to treat SLE [136, 137]. Among them, ALW has a protective effect against lupus nephritis in MRL/lpr mice by reducing glomerular antibody deposition and inhibiting renal proliferation, fibrosis and inflammation [138]. Although small molecular peptides play an important role in immune tolerance and low toxicity, they are also limited in stability and kidney specific targeting, and the existing technology for stable delivery of targets to the kidney is still insufficient. Targeting ALW to specific tissues using nanotechnology is an attractive strategy to enhance drug efficacy and minimize off‐target effects. Wang Y et al. utilized PEG‐PLGA nanoparticles to further modify D‐ALW, demonstrating excellent renal targeting ability and prolonging the peptide's half‐life [62]. This treatment significantly reduced the deposition of IgG and C3 in the glomeruli of lupus mice, and improved renal histopathology, including glomerular proliferation and inflammatory cell infiltration. Furthermore, inflammation and tissue damage in SLE are related to the switching of autoantibody classes and complement activation. Shirai et al. used IgD cross‐linked coupler to pair six anti‐IgD mAbs to dextran in MRL /lpr lupus mice. Treatment with dextran conjugate reduced serum IgG antibodies against ds‐DNA and histones by 50%, compared to PBS control [107]. Hasebroock et al. detected tissue‐bound iC3b/C3d by attaching supermagnetic iron oxide particles (SPIO) to CR2's iC3b/C3d binding region and monitored disease activity in MRL/lpr mice [87].

### 7.6 Targeting Renal Intrinsic Cells

A variety of intrinsic cells in renal tissue are also involved in LN progression, including mesangial cells, podocytes, GECs, and tubule epithelial cells. Drug delivery targets to renal intrinsic cells may play a crucial role in LN kidney injury progression and acute/chronic inflammation. The identification of drug delivery targets within renal intrinsic cells may hold a pivotal role in the treatment of LN‐induced kidney injury, as well as in the manifestation of acute and chronic inflammatory responses. The complexity of the renal structure and inherent cells provides targets for nanoparticle‐based drug delivery to the kidney. Nanocarrier materials used for drug administration primarily encompass active targeting, passive targeting, and other mechanisms specific to the kidney.

The kidney possesses fenestrated endothelium, which permits the passive accumulation of nanoparticles (NPs), making it an accessible site for NP delivery. In the glomerular filtration barrier, glomerular capillary openings (60–80 nm) and slit diaphragms (12–22 nm) at podocyte foot processes constitute a physical barrier for hemofiltration, with particle size influencing renal drug delivery [139, 140]. Particles <10 nm in diameter typically pass freely into renal tubules, unimpeded by glomerular filtration [141]. Particles in the size range >10 and <200 nm may encounter obstruction at the opening of the podocyte foot process or glomerular basement membrane. Pegylated gold NPs smaller than 100 nm have been reported to accumulate in the renal mesangium [142].

By incorporating specific functional groups or bioactive molecules, such as receptor ligands for tubule cells and glomerular proteins, on the nanomaterial surface, the nanoparticles exhibit enhanced affinity and targeting capabilities, facilitating efficient kidney tissue interaction. Maeda K et al. developed antipodocin/antirenin‐tagged nanogel encapsulating CaMK4 inhibitor KN93, mitigating podocyte damage and renal inflammation in lupus mice [108]. Integrin receptors are expressed on the surface of mesangial cells, and these integrins play a crucial role in glomerular development and interaction with extracellular matrix proteins. Scindia Y et al. found that *α*8 integrin could be used as a potential target for drug delivery in nephrotic mice, and constructed an anti‐*α*8 integrin immune liposome to achieve specific delivery of mesangial tissue in lupus mice [109]. Tuffin G et al. utilized mesangial cells to express Thy1.1 antigen and developed Thy1.1‐targeting Fab fragments for liposome binding, forming OX7‐conjugated immune liposomes (OX7‐IL) that successfully deposited drugs in glomeruli and colocalized with mesangial cell markers [110]. E‐selectin is expressed on GECs during the onset of glomerulonephritis in mice. Asgeirsdottir SA et al. constructed liposomes binding anti‐e‐selectin antibodies (AbEsel liposomes) to successfully target dexamethasone to GECs [111]. Many studies also indicate that exosomes have the potential to target kidney innate cells. As the initial barrier of the glomerular filtration membrane, GECs are more prone to inflammation, proteins, lipids, and other biomolecules. Wang B et al. showed that miR‐let‐7c from MSCs was be delivered to mouse renal epithelial cells via exosomes [112]. Chen J et al. found that when Lyme‐Exos derived from spleen lymphocytes transmit information to the kidney over a long distance, GECs were likely to be one of the main cells involved in this process [113]. Funahashi Y et al. found a kidney‐specific nanoparticle enabling specific within‐kidney targeting to proximal tubular epithelial cells provided by the megalin ligand cilastatin to improve the safety and efficacy of dexamethasone [114].

## 8 Discussion

### 8.1 Challenges and Prospects

With the ongoing evolution of nanotechnology, nanomedicine emerges as a promising avenue to propel targeted drug therapy to the pinnacle of disease treatment. This potential stems from nanomedicine's modular platform, which facilitates its ease of personalization, customization, and modification specifically for therapeutic agents. For instance, the administration of nanomedicine possessing superior biocompatibility and biodegradability can significantly enhance the efficacy of drugs both in vivo and in vitro, without the concern of potential adverse effects. In the study of SLE/LN, the drug therapy based on nanomaterials has received more and more attention. This article reviews the research progress of nanomaterial‐based drug therapy for SLE\LN, aiming to reveal the role of different nanomaterials and drug delivery targets in SLE/LN therapy and further to elucidate their immunomodulatory mechanisms. SLE is a chronic, multisystem autoimmune disorder characterized by an array of severe complications, including Lupus Nephritis (LN). Chronic inflammation serves as a fundamental driver in the pathogenesis of SLE. Consequently, the capacity to effectively manage this inflammatory process is paramount in mitigating the progression of associated diseases. Over the past several decades, pharmacological interventions aimed at managing SLE have witnessed substantial advancements. Nevertheless, contemporary therapeutic drugs for SLE and LN are associated with several drawbacks, including limited solubility, abbreviated circulating half‐lives, nonspecific tissue distribution, and intrinsic toxicity. There are also many questions about how nanomaterials interact with immune system components, which still require more in‐depth research and deep understanding. The targeted delivery of drugs to immune cells through nanomaterials is of great significance for SLE/LN immunotherapy. The optimal drug delivery system for SLE and LN involves targeting overactivated immune cells, including T cells, B cells, dendritic cells, and macrophages, as well as sites of renal pathology such as IC deposits, endothelial cells, and mesangial cells. Cellular immunotherapy holds great promise as a therapeutic modality for various diseases, yet it is hampered by several disadvantages, notably including limited drug targeting, reduced half‐life, and suboptimal absorption rates. Nanomedicine, emerging as an innovative drug delivery platform, presents viable solutions to these challenges. Drugs including nucleic acids, proteins and small molecules can be efficiently delivered to the target site of cellular immunotherapy by nanosystems.

First, ligands are meticulously designed to adhere to the surfaces of nanomaterials, specifically anti‐CD3/CD28 antibodies for targeting T cells, anti‐CD22 antibodies for B cells, anti‐CD11C antibodies for dendritic cells, and rHDLs for macrophages. Secondly, novel nanomaterials with specific targeting capabilities. are designed to target different cells. For instance, P2NS‐luteic acid (GA) specifically targets CD71+ immune cells, a linear aliphatic polyester based on lactic acid structure targets B cells, P‐D2 peptide lipid structure‐density‐modified polymers are designed to target CD11c+ dendritic cells, and ursodeoxycholic acid (pUDCA) NPs precisely target TGR5+ monocytes and macrophages. LN, a renal manifestation of SLE, involves passive and active nanomaterial transport to specific renal intrinsic cells. The kidney is a fluid filtration barrier system, and the passive targeting of nanoparticles to the kidney is related to particle size, surface charge, particle shape, and so on. Kidney active transport entails incorporating specific functional groups or bioactive molecules, like renal tubule cell receptor ligands and glomerular proteins (e.g., anti‐podocin, anti‐renin for podocytes; anti‐*α*8 integrin for mesangial cells; anti‐e‐selectin for endothelial cells), onto nanomaterial surfaces. This enhances nanoparticle affinity and targeting, enabling efficient kidney tissue interaction.

In conclusion, nanomedicine can effectively address targeting issues in cellular immunotherapy and enhance its efficacy. However, the manifestation of excessive immune responses in SLE/LN via various immune cell subsets is intricate, encompassing the dysregulation between Th1, Th2, Th17, and Tfh cells, as well as the imbalance between resident macrophages and monocytes‐derived macrophages. However, current studies often design nanomaterials solely for common immune cell subsets, including T cells, B cells, and macrophages. Therefore, the potential to design nano‐loaded drug materials that exhibit enhanced targeting to specific immune cell subgroups holds promise for further advancing the clinical application of nano‐targeted material‐mediated cellular immunotherapy. Such precision in targeting would significantly propel the field toward more effective and tailored therapeutic interventions.

Furthermore, neutrophils constitute a vital immune cell population in SLE/LN, playing a pivotal role in disease initiation. The dysregulation of neutrophils may exert a distinct and crucial pathogenic influence in the disease progression. Circulating neutrophils can enter the Fc portion of deposited ICs through open pores in the glomerular capillary endothelium and activate the associated activated complement components, making the kidneys particularly vulnerable to neutrophil‐mediated inflammation and subsequent damage. However, there is currently no therapeutic approach that can inhibit neutrophil responses during renal inflammation. A deeper understanding of the role of neutrophils and precise targeting of their recruitment to the kidneys will pave the way for therapeutic strategies to regulate renal inflammatory responses in LN. By designing a nanomaterial drug delivery system that specifically targets neutrophils, we can achieve inhibition of neutrophil recruitment at the renal level without affecting other systems, thereby minimizing the risk of infection and malignant tumors while modulating the immune system to alleviate and treat renal damage. However, there is currently limited research on nanomaterials targeting neutrophils in SLE/LN, which merits further exploration and represents a new challenge.

Although nanomaterial‐based therapies show promising potential in preclinical trials for SLE/lupus nephritis, their clinical translation faces both conceptual and practical hurdles. Their advantages, such as enhanced targeting specificity, reduced systemic toxicity of encapsulated drugs (e.g., dexamethasone prodrugs), and potential for combination therapies, are accompanied by certain limitations [143]. These include the manufacturing complexity of biomimetic nanomaterials (e.g., exosomes, cell membrane‐coated nanoparticles), which lack standardized protocols; concerns regarding the immunogenicity and toxicity of certain inorganic materials (e.g., gold, iron oxide); and the need for more precise targeting systems tailored to specific immune cell subsets and renal cells involved in disease pathogenesis [144]. Furthermore, the heterogeneity of SLE/lupus nephritis necessitates personalized, immunophenotype‐based approaches, as a universal nanomedicine may have limited applicability [145]. Recent advances, including stimuli‐responsive designs that adapt to pathological microenvironments and multivalent targeting platforms for enhanced specificity, indicate a trend toward more intelligent and adaptable nanotherapeutics. Future work must prioritize rigorous pharmacokinetic studies, long‐term safety assessments, and the development of drug delivery systems informed by patient stratification [146, 147].

### 8.2 Safety and Barriers

The clinical translation of nanomedicines requires rigorous safety evaluation, with primary concerns including nontargeted accumulation and clearance—where nanoparticles smaller than 10 nm are rapidly cleared by the kidneys but may accumulate in renal tubules causing potential nephrotoxicity, while those larger than 100 nm or with hydrophobic surfaces are more readily captured by macrophages in the liver and spleen (e.g., Kupffer cells), reducing drug delivery efficiency and potentially leading to hepatosplenic toxicity or prolonged immune activation [148]; immunogenicity and allergic reactions—as certain nanomaterials, particularly some cationic polymers or liposomes, can activate the complement system, triggering complement activation‐related pseudoallergy (CARPA) manifesting as infusion reactions that may be severe [149, 150], and the widespread use of polyethylene glycol (PEG) carries the risk of inducing anti‐PEG antibodies, potentially causing accelerated blood clearance and allergic reactions upon repeated dosing [149]; and long‐term biocompatibility—where the long‐term fate, degradation pathways, and potential chronic toxicity of inorganic nanomaterials (e.g., gold, iron oxide) require further investigation. Addressing these challenges necessitates a “safety‐by‐design” approach: optimizing physicochemical properties (size, charge, hydrophilicity), incorporating “stealth” coatings (e.g., CD47‐mimicking peptides to avoid phagocytosis), and potentially screening patients for complement activation propensity or pre‐existing anti‐PEG antibodies [151].

## 9 Conclusion

The convergence of nanotechnology and immunology offers revolutionary potential for SLE/LN therapeutics through precision‐targeted nano‐delivery systems. Current progress in renal microenvironment‐responsive nanocarriers with controlled drug release profiles highlights the need for optimized biocompatibility and biological barrier penetration. Future research must prioritize rationally engineered dual‐targeting platforms addressing immune dysregulation and renal parenchymal injury, establishing robust preclinical models for clinical translation of nano‐immunotherapy in autoimmune nephropathies.

## Author Contributions

**Cheng Zhou:** conceptualization (lead), data curation (lead), formal analysis (lead), writing – original draft (lead), writing – review and editing (equal). **Jiayi Li:** formal analysis (equal), methodology (supporting), writing – original draft (supporting). **Jian Zhang:** data curation (equal), validation (equal). **Haifeng Wang:** validation (lead), visualization (equal). **Haitao Lu:** conceptualization (equal), writing – review and editing (equal). **Shunlai Shang:** conceptualization (equal), funding acquisition (lead), project administration (equal), resources (lead), writing – original draft (equal), writing – review and editing (equal). **Wenge Li:** conceptualization (equal), project administration (lead), writing – original draft (equal), writing – review and editing (lead).

## Funding

This study was supported by National High Level Hospital Clinical Research Funding (2024‐NHLHCRF‐JBGS‐WZ‐03 and 2023‐NHLHCRFYS‐01), Young Elite Scientists Sponsorship Program by CAST (2023QNRC001), Elite Medical Professionals Project of China‐Japan Friendship Hospital (ZRJY2023‐GG06), Cross‐sectional project of ChinaJapan Friendship Hospital (2023‐HX‐103), The Open Grant from the Pingyuan Laboratory (2023PYOP‐0203), Beijing Natural Science Foundation (7244407) and National Natural Science Foundation (82400846 and 82274327).

## Conflicts of Interest

The authors declare that they have no known competing financial interests or personal relationships that could have appeared to influence the work reported in this paper.
